# Explicit neural signals reflecting reward uncertainty

**DOI:** 10.1098/rstb.2008.0152

**Published:** 2008-10-01

**Authors:** Wolfram Schultz, Kerstin Preuschoff, Colin Camerer, Ming Hsu, Christopher D Fiorillo, Philippe N Tobler, Peter Bossaerts

**Affiliations:** 1 Department of Physiology, Development and Neuroscience, University of CambridgeDowning Street, Cambridge CB2 3DY, UK; 2 Division of Humanities and Social Sciences, California Institute of TechnologyPasadena, CA 91125, USA; 3 Department of Neurobiology, Stanford UniversityStanford, CA 94305, USA; 4 Laboratory for Decision Making under Uncertainty, Ecole Polytechnique Fédérale de Lausanne1015 Lausanne, Switzerland

**Keywords:** probability, risk, ambiguity, dopamine, striatum, orbitofrontal cortex

## Abstract

The acknowledged importance of uncertainty in economic decision making has stimulated the search for neural signals that could influence learning and inform decision mechanisms. Current views distinguish two forms of uncertainty, namely risk and ambiguity, depending on whether the probability distributions of outcomes are known or unknown. Behavioural neurophysiological studies on dopamine neurons revealed a risk signal, which covaried with the standard deviation or variance of the magnitude of juice rewards and occurred separately from reward value coding. Human imaging studies identified similarly distinct risk signals for monetary rewards in the striatum and orbitofrontal cortex (OFC), thus fulfilling a requirement for the mean variance approach of economic decision theory. The orbitofrontal risk signal covaried with individual risk attitudes, possibly explaining individual differences in risk perception and risky decision making. Ambiguous gambles with incomplete probabilistic information induced stronger brain signals than risky gambles in OFC and amygdala, suggesting that the brain's reward system signals the partial lack of information. The brain can use the uncertainty signals to assess the uncertainty of rewards, influence learning, modulate the value of uncertain rewards and make appropriate behavioural choices between only partly known options.

## 1. Introduction

Every day we make decisions about the goals we like to pursue, but we do not even know how the brain processes the simplest parameters that determine our decisions. Blaise Pascal 350 years ago employed the emerging probability theory to postulate a formal description of decision making. Outcomes of our choices have specific magnitudes and occur with specific probabilities. Therefore, they can be adequately described by probability distributions of outcome magnitudes. Pascal conjectured that humans tend to select the option whose probability distribution has the highest expected (mean) value compared with all other options. However, choice behaviour is also known to depend on uncertainty, which refers to the width or spread of the probability distribution. Experimental economic and behavioural ecological studies have confirmed that uncertainty is ubiquitous, influences learning and contributes crucially to the valuation of options during decision making in such diverse situations as animals engaging in foraging, ducks distributing proportionally to food sources and bees choosing among different flowers, people deciding between exploration and exploitation and buying into stock markets, companies pricing insurance, and countries evaluating financial, military, social and environmental risks (McNamara & Houston 1980; Harper 1982; Stephens & Krebs 1986; Real 1991; Sutton & Barto 1998; Holt & Laury 2002; McCoy *et al*. 2003; Bossaerts & Plott 2004; Weber *et al*. 2004). Thus, the decision maker needs to evaluate both the expected outcome values and the uncertainty associated with the options. Attentional learning rules, which provide better descriptions of learning in some situations, propose that learning is monotonically related to stimulus-driven forms of attention that vary as a function of uncertainty about reinforcers (Mackintosh 1975; Pearce & Hall 1980). Thus, the abundance of uncertainty in the physical and biological world is widespread and has substantial, often crucial, impact on choice behaviour and learning. These arguments make the investigation of neural mechanisms of uncertainty an important research topic.

### (a) Risk and ambiguity as forms of uncertainty

In theories of choice under uncertainty used in social sciences and behavioural ecology, the only variables that should influence a choice are the judged probabilities of possible outcomes and the evaluation of those outcomes. However, the choices can vary greatly in the level of information available to the decision maker. The probability distributions of outcomes are not always fully known, and confidence in judged probability can vary widely. In some choices, such as gambling on a roulette wheel, probability can be confidently judged from relative frequencies, event histories or an accepted theory. At the other extreme, such as in weather forecasts for distant tourist destinations, probabilities are based on meagre or conflicting evidence, where important information is clearly missing. These two forms of uncertainty are often called risky and ambiguous, respectively. Standard expected utility theory, however, precludes agents from acting differently in the face of risk and ambiguity: even when probabilities are unknown, the agent may still assign probabilities to all possible events before making decisions; otherwise inconsistencies will affect the agent's decisions (Ellsberg 1961). Competing theories view risk and ambiguity as two extremes of a continuum of uncertainty or as two distinct forms of uncertainty with possibly separate underlying neural systems. It is noted that decision makers often have only partial and changing information about probabilities and thus operate by definition on ambiguous outcomes until probabilities are fully established and the definition of risk is fulfilled.

Risk denotes the degree of uncertainty inherent in known probability distributions and can, in the first degree, be expressed as variance (second moment of probability distribution) or its square root, the standard deviation (Markowitz 1952). Variance reflects the spread of a distribution and indicates how far possible values are away from the mathematical expectation of value (expected value, the ‘mean’ of the probability distribution of values, defined as the sum of values multiplied by their respective probabilities). Intuitively, ‘risk’ denotes how much a decision maker in uncertain situations risks to gain or lose relative to the known mean possible outcome (expected value of the known probability distribution). Probability itself is not a monotonic measure for risk. For example, in a two-outcome situation such as reward versus no reward, outcome value increases linearly with the probability of outcome, whereas risk is maximal at *p*=0.5 and decreases towards higher and lower probabilities as it becomes increasingly certain that something or nothing will be obtained (figure 1).

Ambiguity, in contrast to risk, refers to situations of uncertainty in which we have only incomplete information about the probability distributions. This occurs typically when making weather predictions in regions of the world we are not familiar with or betting in games whose rules we fail to understand. In controlled laboratory settings, ambiguity as opposed to risk can be tested quantitatively in conditions of uncertainty by withholding parts of information about probabilities.

Economic decision theories, such as expected utility theory and prospect theory, build on the basic terms of expected value and uncertainty and incorporate them into the scalar decision variables of expected utility and prospect, respectively (Von Neumann & Morgenstern 1944; Kahneman & Tversky 1979). Utility is defined as the subjective value we design to objective outcome values; it is measured in an objective manner by behavioural preferences. Expected utility refers to the mean of the probability distribution of utilities, defined as the sum of utilities multiplied by their respective probabilities. Many decision makers often show gradually flattening, concave utility functions, indicating that the gains achieved by ever higher outcomes become gradually less important. This decreasing marginal utility leads to the aversion of risky outcomes, as the potential losses loom larger than the gains. However, behavioural attitudes towards uncertainty are not identical across individuals and are not even constant within the same individuals, as shown in animal foraging (Caraco *et al*. 1980, 1990) and human risk assessments (Weber & Milliman 1997). During risk seeking, decision makers assign increasingly greater utility to higher outcomes and show convex utility functions. The gains from larger than mean outcomes more than offset the losses incurred by smaller than mean outcomes, thus encouraging the choice of risky options. Thus uncertainty influences the valuation of outcomes, and expected utility is not only determined by the expected value of outcomes but also by their variance. The dependence of expected utility on variance is captured mathematically by the Taylor series expansion of expected utility, which separates the mathematical expectations of value (first moment) from variance (second moment) and higher moments. This is conceptualized in the mean variance approach of financial decision theory and foraging theory (Levy & Markowitz 1979; Stephens & Krebs 1986; Huang & Litzenberger 1988). Ambiguity might have a similar, and even stronger, influence on expected utility compared with risk. Risk-averse people are typically more willing to bet on risky rather than on ambiguous outcomes, indicating an even stronger aversion for ambiguity compared with risk due. Taken together, the scalar variable of expected utility appears to be composed of two distinct entities, the expected value and the uncertainty in the form of risk or ambiguity.

### (b) The reward system and uncertainty

A basic issue in neuroeconomics concerns the neural processing of key decision variables and the brain mechanisms underlying decision making under uncertainty. Given that expected value and uncertainty constitute basic decision variables, it is reasonable to ask how these variables are processed in the brain. Electrophysiological studies have identified the brain's reward system as a restricted network of structures, which include the dopamine neurons of the pars compacta of substantia nigra and ventral tegmental area, the striatum, orbitofrontal cortex (OFC) and amygdala (Cromwell & Schultz 2003; Fiorillo *et al*. 2003; Tobler *et al*. 2005; Padoa-Schioppa & Assad 2006; Paton *et al*. 2006). The pure reward signals in these structures encode reward value as magnitude or probability of reward irrespective of other sensory or motor attributes. In addition, expected reward influences movement-related activity in the parietal cortex, dorsolateral prefrontal cortex, anterior and posterior cingulate cortex and striatum (Watanabe 1996; Platt & Glimcher 1999; Shidara & Richmond 2002; Cromwell & Schultz 2003; McCoy *et al*. 2003; Musallam *et al*. 2004; Samejima *et al*. 2005). Human neuroimaging studies found regional activations related to expected reward value in similar brain structures, including the striatum, globus pallidus, midbrain, medial prefrontal cortex, OFC and anterior cingulate cortex (Knutson *et al*. 2005; Preuschoff *et al*. 2006; Tobler *et al*. 2007). Some of these regional activations may be due to inputs from dopamine reward signals. Thus, expected value as a key economic decision variable appears to be encoded by neurons in the brain's reward system.

The rationale for investigating risk and ambiguity derives from several considerations.

- The ubiquitous uncertainty about outcomes of behaviour needs to be detected and assessed by individuals in order to gain an accurate perception of the environment. Different forms and degrees of uncertainty, such as risk and ambiguity, should be processed as separate or quantitatively different signals to optimize their detection and discrimination, irrespective of their use for immediate behavioural choices.
- The mean variance approach of financial economics (Levy & Markowitz 1979) postulates that the first two moments of probability distributions, expected value and variance, are assessed separately and are combined in a flexible and adaptive manner to represent the expected utility of all available outcome options. By contrast, alternative decision theories, such as the expected utility framework, do not require the combination of the first two moments but calculate the expected utility as the sum of the probability-weighted scalar utilities of all outcomes. The combination of value and uncertainty signals, or the singular expected utility signal, would provide direct information and explicit direction for overt choices. Our current data lend support to the mean–variance approach of utility and therefore will be cast in these terms. However, by describing these data, we do not exclude the possible existence of neural signals coding utility as a scalar variable.
- Magnitude, probability, expected value or uncertainty might be misrepresented in the brain or inappropriately integrated into a utility signal and thus provide false inputs to neural mechanisms involved in choices. Such distorted choice signals, or their distorted influences during decision making, might contribute to paradoxical choices, such as seen in preference reversals, which are not covered by standard expected utility theory and have given rise to prospect theory. It might be that particular neural signals in the brains of individual decision makers, rather than market or other external forces, induce the often detrimental paradoxes of choices. Finding a potential neural basis for anomalous economic choices would be analogous to using the specific properties of neural signals in the visual cortex for explaining illusory perceptions (Livingstone & Hubel 1988). To unravel biological mechanisms, underlying paradoxical economic decisions would be a major achievement of neuroeconomic studies.

### (c) Scope of the review

This review addresses the issue of how uncertainty as a key determinant of economic choices and a modulator of learning gives rise to explicit signals in the reward system of the brain. We present studies designed specifically to investigate how reward uncertainty might be encoded in the neural and metabolic activity of the brain. We describe initial electrophysiological studies that revealed risk signals in single neurons and human imaging studies that built partly on these studies but went beyond to identify distinct brain structures coding different forms of uncertainty, even in relation to risk attitudes of individuals. We believe that these uncertainty signals represent discrete neural events that would be useful for the perception of uncertain environments and for making decisions under uncertainty. All reviewed studies use predominantly Pavlovian reward predictors, sometimes overlaid onto operant responses, and the studies were not designed to contribute to the distinction between goal-directed and habit behaviours. Despite the focus on the reward system, we do not suggest that uncertainty coding occurs primarily for rewards. Other functional brain systems have simply been less well investigated, with notable exceptions (e.g. Basso & Wurtz 1997).

## 2. Risk signals in single neurons

### (a) Coding of risk in dopamine neurons

The first two moments of a Gaussian probability distribution, expected value and variance, can be used to distinguish value from risk of reward. Reward value can be expressed as the mathematical expectation of reward. In the case of only two possible reward outcomes, expected value increases monotonically with the probability of the higher outcome, whereas risk expressed as standard deviation or variance follows an inverted U-shaped function of probability, increasing towards *p*=0.5 and declining thereafter (figure 1). Entropy shows a similar inverted U function, its maximum being 1 bit at *p*=0.5.

We trained two macaque monkeys (*Macaca mulatta*) in a Pavlovian task without choice, in which a specific visual stimulus on a computer screen indicated the probability of receiving after 2 s a drop of fruit juice of fixed magnitude of approximately 0.15 ml (Fiorillo *et al*. 2003). Employed probabilities were *p*=0, 0.25, 0.5, 0.75 and 1.0. Thus, each stimulus indicated a specific probability distribution with two elements, 0 and 0.15 ml. Anticipatory licking responses during the interval between stimulus and reward increased with the probability of reward, indicating that the animals discriminated the stimuli behaviourally according to expected reward value. We used standard electrophysiological methods and criteria to record extracellularly the impulse activity of single dopamine neurons in groups A8, A9 and A10 of the substantia nigra pars compacta and the medially adjoining ventral tegmental area in the ventroanterior midbrain.

The majority of dopamine neurons showed transient responses of impulse activity (activations) to the reward-predicting stimuli that increased monotonically with reward probability (Fiorillo *et al*. 2003). Additional variations in reward magnitude showed that the dopamine responses encoded the expected value (mean) of reward (Tobler *et al*. 2005). The activations following the reward itself decreased monotonically with increasing probability, and the depressions with reward omission increased with probability, thus reflecting quantitative relationships of the known reward prediction error coding (Schultz *et al*. 1997). These dopamine signals apparently encode the value of rewards as defined by reward probabilities.

At least one-third of dopamine neurons showed an additional, separate, slower and more sustained activation during the interval between the stimulus and the reward which tended to increase as the interval elapsed. The signal was the highest at *p*=0.5 and lower at lower and higher probabilities (figure 2). Owing to this inverted U-shaped relationship to probability, the signal correlated best with risk but not with the expected (mean) value of reward. Whereas the above experiment varied the probability of reward of a specific magnitude, an additional test used distinct conditioned stimuli, each predicting two different, non-zero reward magnitudes, each delivered with a probability of 0.5. Risk was measured as standard deviation or variance of these distributions. As in the previous experiment, the sustained activation between stimulus and reward increased with the risk of reward outcomes. The risk signal occurred in the same population of dopamine neurons that encoded reward value but was uncorrelated with the more phasic value responses, which increased monotonically with probability. Thus the slow, sustained dopamine signal apparently encoded the risk of rewarding outcomes.

Taken together, dopamine neurons encode at different time points two fundamentally distinct pieces of information about reward outcomes. The phasic signals to stimuli and reward carry information about reward value prediction and error, whereas the more sustained signal encodes reward risk. The dopamine risk signal could provide an input to brain structures dealing with the assessment of reward risk *per se*. Furthermore, it could combine with a reward value signal, even in the same dopamine neurons, to represent information about the expected utility in risk-sensitive individuals, according to the mean variance concept in financial decision theory (Levy & Markowitz 1979).

### (b) Influence of risk on cortical movement-related activity

A recent study employing an oculomotor choice task described a risk signal in the posterior cingulate cortex (McCoy & Platt 2005). As in one of the dopamine experiments, the study employed binary reward distributions with two equiprobable (*p*=0.5) reward magnitudes and different standard deviations. Cingulate neurons showed increased activations related to saccadic eye movements as the risk in the choices increased. These data suggest the coding of outcome risk during behavioural choices. The cortical signal could provide essential information for assessing the subjective preferences among rewards with different utilities when making decisions under conditions of risk.

## 3. Risk and ambiguity signals in human brain structures

### (a) Coding of risk

The experiments followed the rationale of the recordings from dopamine neurons and used variations in the probability of fixed reward outcomes to assess brain responses to risk separately from reward value. As with dopamine neurons, the task design distinguished between expected reward value, which increased monotonically with probability, and risk, which varied as an inverted U function of probability and was the highest at *p*=0.5 and decreased towards lower and higher probabilities (figure 1). Rewards were fictive money units. Functional magnetic resonance imaging (fMRI) served to measure human blood oxygenation levels in response to specific stimuli predicting reward outcomes with specific value and risk (blood oxygen level-dependent (BOLD), responses).

One experiment used a card task in which human participants were presented with two successive cards containing a number between 1 and 10 (Preuschoff *et al*. 2006). Before the first card was shown, the participants placed a bet on which of the two cards would be higher. Thus, the presentation of the first card indicated the probability of receiving a reward, ranging from *p*=0.0 to 1.0 in steps of 0.1, and presentation of the second card indicated whether a money reward was won or not. For instance, if the subject bet on ‘second card higher’, the probability of winning was given by the number of cards initially in the deck (always 10) minus the number displayed on the first card (*C*) and divided by the number of cards remaining in the deck: *p*=(10−*C*)/9. Motivation and stimulus salience were assessed by measuring the reaction time to detection of the second card and failed to covary with risk, thus ruling out these simple confounds of risk coding.

Using the card task, we assessed first the coding of expected reward value as monotonic increases of BOLD responses with increasing probabilities (Preuschoff *et al*. 2006). Regressions using a general linear model for expected reward value revealed significant BOLD responses during the initial 1 s following presentation of the first card in putamen, ventral striatum, globus pallidus, anterior cingulate cortex, midbrain and a few other regions. The BOLD responses in the ventral striatum and putamen on both sides increased monotomically across the 10 reward probabilities that arose as a result of the number on the first card (*r*^2^'s 0.66–0.87). These data obtained with variations in probability confirm the coding of expected value in the striatum shown previously with reward magnitude (Delgado *et al*. 2000; Elliott *et al*. 2003; Knutson *et al*. 2003).

A second experiment was based on the same, monotonic versus inverted U distinction between value and risk and used specific visual pictures, each of which predicting a specific reward magnitude and probability (*p*=0.0–1.0 in steps of 0.25; Tobler *et al*. 2007). Expected value was tested by varying both magnitude and probability of reward. BOLD responses to the stimuli increased monotonically with predicted reward magnitude and probability in the medial and ventral striatum. Although some parts of the striatum encoded magnitude and probability in separation, an overlapping region in the medial striatum showed monotonic increases with both measures of reward value, thus encoding value irrespective of the underlying combination of magnitude and probability. These striatal regions overlapped with those coding reward probability in the first experiment (Preuschoff *et al*. 2006).

We investigated the coding of risk in the card task and aimed to reveal a relatively tonic risk signal similar to that seen in dopamine neurons during the period between stimulus and reward (Preuschoff *et al*. 2006). The general linear model assumed this time course and tested risk coding as inverted U function of probability during 6 s between the first probability-predicting card and the second reward-indicating card. The regression revealed activations in an area extending posterior to and bilateral from the ventral striatum to the subthalamic nucleus as well as mediodorsal thalamic nucleus, midbrain and bilateral anterior insula (figure 3*a*). Subsequent regressions on the slope coefficient beta of the general linear model revealed significant correlations of BOLD responses with risk as an inverted U function across all probabilities in the ventral striatum on both sides, midbrain and thalamus (figure 3*b*; *r*^2^'s 0.80–0.89). Interestingly, reward probability was uniformly *p*=0.5 during the initial placement of the bet before the first card. Regression of BOLD responses during this period was significant, and betas were within the same range as with activations between the two cards at *p*=0.5 (grey dots in figure 3*b*). A separate activation in the anterior insula covaried with the difference between the actual risk informed by each card and its prediction (risk prediction error).

The ventral striatum showed an interesting time-dependent conjunction of value and risk coding (Preuschoff *et al*. 2006). We mapped the BOLD responses for the expected reward during the initial 1 s period following the first card, together with risk during the 6 s period following the first card. We found an overlapping region in the left ventral striatum in which the BOLD response covaried early with expected reward increasing monotonically with probability but subsequently reflected the risk following an inverted U function of probability.

We assessed risk also in the picture task, using the scheme of inverted U function of probability (Tobler *et al*. 2007). We regressed a more phasic response of 2.5 s duration following the reward probability predicting stimuli and found that BOLD responses to the pictures increased with risk in the lateral OFC. The activations correlated with variance but not expected value, indicating a distinct risk signal in the OFC. The OFC was not explored for a more tonic risk signal in the first study (Preuschoff *et al*. 2006).

Taken together, humans show risk signals in the ventral striatum, midbrain, anterior insula and OFC. The risk signals are spatially well separated from reward value signals and thus occur in different neurons, or they show at least different time courses in similar ventral striatal regions. The data obtained with the card task suggest relatively slow risk signals in human brain structures that receive dopamine afferents, including the ventral striatum, and might reflect input from the similar risk signal seen in dopamine neurons. The more rapid risk signal in the OFC might be distinct from the slower ones found in the striatum and associated structures, potentially suggesting that different risk signals with different time courses occur in separate brain structures. The results demonstrate that human risk signals can be investigated with BOLD responses based on the mean variance concept in financial decision theory, which separates outcome value from risk.

### (b) Covariation of risk signals with individual risk attitudes

We used a choice version of the picture task to assess individual risk attitudes (Tobler *et al*. 2007). Individual participants chose between a safe and a risky gamble with the same expected value. The risky gamble produced one of two equiprobable (*p*=0.5) reward magnitudes. We assessed the individual attitudes towards risk in a choice task between safe and risky outcomes. Each time a participant preferred the safe option, the score of risk aversion increased by 1, whereas choosing the risky option decreased it by 1 (four choices). A positive total score indicated risk aversion, a negative score risk seeking and a zero score risk neutrality.

We regressed the goodness of fit of the risk signals in all the participants against their individual risk aversion scores. We found a risk signal in the lateral OFC that increased with the degree of risk aversion, whereas a risk signal in a more medial part of OFC increased with risk seeking (figure 4). In addition, a region in the anterior superior frontal gyrus showed a decreasing risk signal only in risk-averse participants, whereas a region in the caudal inferior frontal gyrus showed an increasing risk signal only in risk seekers.

These data suggest that risk signals are not the same across different individuals but vary according to individual risk attitudes. The individual variations in risk signals may explain the different attitudes of individuals towards risk and influence their decision making in risky situations.

### (c) Coding of ambiguity

Ambiguity refers to the form of uncertainty in which outcome probabilities are incompletely known, as opposed to risk where probabilities are known. Uncertainty-averse individuals often express pessimism over ambiguous outcomes in being more averse to ambiguity than to risk; they prefer risky over ambiguous gambles, indicating an inverse relationship between the utility of outcomes and the degree of knowledge about probabilities. Ambiguity can lead to inconsistent choices and preference reversals; it could be viewed as a more profound form of uncertainty compared with risk, with stronger impact on behavioural preferences.

We used choices between certain and uncertain monetary outcomes in three situations in which the uncertain option dissociated ambiguity from risk based on different amounts of information (Hsu *et al*. 2005): (i) in the card deck situation, the uncertain option involved either a risky gamble where probabilities were known or an ambiguous option with only partly known probabilities. (ii) The knowledge situation modelled a more cognitive choice task in which the uncertain options involved events and facts that fell along a spectrum from risk to ambiguity, such as temperature judgments for more (risk) or less well-known cities (ambiguous). (iii) The informed opponent situation involved bets of the participant against another person who has seen a sample of cards from the deck. This opponent is therefore better informed about the contents of the ambiguous deck. This condition corresponds to a commonly posited theory of ambiguity aversion: even when there is no informed opponent, people act as if there is one.

The human fMRI study aimed to identify neural ambiguity signals by dissociating between the ambiguous and risky situations. We used two primary regressors, one for the safe versus ambiguous choice and one for the safe versus risky choice, and applied them to a task period between the onset of the stimulus and the time of choice. In the three experimental situations pooled, BOLD responses were higher for ambiguous gambles compared with risky ones in the OFC (figure 5), amygdala and dorsomedial prefrontal cortex on both sides. The contrast values between ambiguity and risk were positively correlated with the degree of ambiguity aversion in the right and left OFC (*r*'s 0.37–0.55).

In contrast to the ambiguity signals, we found a risk signal in the dorsomedial striatum (caudate nucleus) where BOLD responses were higher for risky compared with ambiguous outcomes (Hsu *et al*. 2005). These striatal activations also correlated with the expected value of actual choices, whereas no such correlation was observed in the OFC or amygdala. The striatal risk signal showed slower time courses with slower build-ups and peaks compared with the ambiguity signals in OFC and amygdala. The difference was present in all three experimental treatments and appeared to be independent of the behavioural choices. Detection of this striatal risk signal corroborates the finding of a risk signal in the medial striatum (Preuschoff *et al*. 2006).

Another study used choices between safe and either ambiguous or risky options comparable to situation (i) above and identified a dissociation between ambiguity and risk signals. Ambiguous gambles induced BOLD responses in the lateral prefrontal cortex that covaried with individual ambiguity attitudes, whereas risky gambles activated the parietal cortex in relation to risk attitudes (Huettel *et al*. 2006).

Taken together, there might be two ways in which ambiguity is coded differently from risk. Some brain structures show stronger BOLD responses to ambiguous compared with risky gambles, such as in parts of frontal cortex and amygdala (Hsu *et al*. 2005). The graded, rather than all or none, differences in uncertainty signals in the same brain structures would be compatible with the idea of a quantitative continuum in uncertainty between risk and ambiguity. It is consistent with a hierarchical Bayes approach to ambiguity. By contrast, other brain structures show specific signals for the two forms of uncertainty that are distributed across mutually exclusive brain structures, notably striatum and parietal cortex (risk) versus parts of frontal cortex and amygdala (ambiguity), consistent with the notion of qualitative differences between risk and ambiguity (Hsu *et al*. 2005; Huettel *et al*. 2006). This separation constitutes a scheme of double dissociation and suggests that these regions process risk and ambiguity as qualitatively different forms of uncertainty.

## 4. Conclusions

The studies reviewed show that reward structures in the human and non-human brains encode basic microeconomic decision parameters and carry separate signals for reward value and uncertainty. Individual dopamine neurons show two different responses to reward value and risk at different time points, respectively, conceivably leading to different temporal profiles of release and synaptic concentration of dopamine. Human BOLD responses, which reflect the metabolic demands of synaptic input activity to specific brain structures (Logothetis *et al*. 2001), demonstrate the separate coding of the (mathematical) expectation of reward value and reward risk (variance) in such dopaminoceptive structures as striatum, insula and OFC, although non-dopaminergic origins of these signals are also possible. The risk signals correlate with individual human risk preferences, suggesting a neural basis for individual variations in risk attitude. From the point of view of financial decision theory, value and risk signals could be components of a neural representation of expected utility. The observed differences in neural signalling for risk and ambiguity might reflect the different degrees of impact these two forms of uncertainty have on the utility of behavioural choice options. Taken together the data suggest largely distinct contributions of reward structures to the coding of value and risk as fundamental parameters of financial decision theory.

Our investigations were guided by the mean variance model of decision making under uncertainty proposed by financial decision theory. This model specifies expectation and variance of reward as the minimal parameters necessary for rational choice under uncertainty in an idealized world with Gaussian distributions. Expected value and risk often change independently and may be balanced against each other. This trade-off has led to important insights into animal foraging behaviour (Caraco *et al*. 1980, 1990; Real 1991) and risk assessment, demand for fixed income securities and pricing of risky securities in humans (Tobin 1958; Weber & Milliman 1997). Experimental tests confirm these predictions (Bossaerts & Plott 2004). Thus, it seems to be advantageous for agents to have independent and sensitive neural signals of expected value and risk which combine dynamically into a neural representation of expected utility according to momentary options and risk attitudes. The currently observed neural value and risk signals could provide exactly such independent pieces of information and could separately contribute to decisions involving risky options. It is striking that brain activation in dopaminoceptive structures reflects the separation of expected reward and risk on which financial decision theory is based.

Our neuronal and imaging studies on risk coding were explicitly conducted under purely perceptual conditions in which no choice was to be made, whereas the ambiguity study involved choices. Many levels of processing intervene between the perception of key decision parameters and an overt behavioural choice. It is likely that the brain tracks expected reward and risk at an initial perceptual level, whereas additional elements downstream from value and risk signals would modulate the final choice, such as contextual factors and decisions by others (Abel 1990). As such, perception of reward and risk may continue even in the absence of choice. Brain activity would reflect primarily the information gathering for the case where a choice opportunity would suddenly arise. By contrast, the BOLD responses to ambiguity occurred during behavioural choices and were stronger when the choices comprised ambiguous compared with risky outcomes. These data confirm that risk and ambiguity signals occur also in choice situations, which appears to validate the hypothesis of perceptual uncertainty signals being carried over into choice situations.

Although we assessed the functions of these brain structures in the context of neuroeconomic experiments, we believe that they subserve general aspects of how organisms explore their environment. Under uncertainty, the brain is alerted to the fact that information is missing, that choices based on the information available therefore carry more unknown (and potentially dangerous) consequences and that cognitive and behavioural resources must be mobilized in order to seek out additional information from the environment.

### (a) Potential functions of dopamine risk signal

The two separate dopamine responses appear to relate to the first two moments of reward probability distributions, namely the phasic reward prediction error signal (expected value), and the slower, more sustained and quantitatively lower ramp (variance, or its square root, standard deviation). Our similarly designed human imaging studies confirm the distinctions between the two signals in the human brain (Preuschoff *et al*. 2006; Tobler *et al*. 2007).

The dopamine risk response could inform neural decision mechanisms on the degree of risk involved in a reward distribution and thus contribute to the known influence of risk on behavioural choices. It could also impact on the normalization of dopamine reward prediction error signal by standard deviation through a neural mechanism of mathematical division (Tobler *et al*. 2005). A normalized error signal would factor out the predicted risk of outcomes and may contribute to stable learning irrespective of risk (Preuschoff & Bossaerts 2007).

The bidirectional coding of reward prediction error by the phasic responses of dopamine neurons follows general principles of learning described by the Rescorla–Wagner (1972) learning rule. The separate dopamine risk signal would covary with the attention induced by risky outcomes and thus might contribute to learning in situations described by the attentional learning rules (Mackintosh 1975; Pearce & Hall 1980). As a possible mechanism, dopamine released by a ramping dopamine risk signal could enhance the dopamine concentration induced by the subsequent phasic reward prediction error signal and thus lead to a stronger effect of dopamine on post-synaptic learning mechanisms, although other, possibly more effective, membrane mechanisms are also conceivable.

### (b) Human risk and ambiguity signals

The search for human risk signals assumed similar slow time courses as found in dopamine neurons. Accordingly, the regressions revealed risk signals in the striatum with relatively late peak latencies of approximately 6 s (Preuschoff *et al*. 2006), which corresponded closely to the time courses of risk signals in the insula and parietal cortex (Huettel *et al*. 2005). Owing to their temporal similarity, these human risk signals might be derived from the risk signal of dopamine neurons. As with dopamine neurons, the slow time courses could serve as a distinction against faster value signals found in the same brain structures, such as specific regions in the ventral striatum in which the initial response reflects expected reward and the subsequent response reflects risk. However, our other studies searched for risk signals with faster time courses and indeed found BOLD responses with shorter peak latencies of approximately 4.0 s in OFC (Tobler *et al*. 2007). These results offer the intriguing possibility that different risk signals occur with different time courses in different brain structures and are driven by different inputs.

Interestingly, the orbitofrontal risk signals were correlated with variations in risk attitude of individual participants (Tobler *et al*. 2007). The lateral OFC showed stronger risk signals with increasing individual risk aversion, whereas medial orbitofrontal activations correlated with increasing risk seeking. Conceivably, these risk structures might show differential overactivity or underactivity in different individuals. As financial decision theory postulates, risk influences expected utility in risk-sensitive individuals, and variations in risk signals between individuals might influence the valuation of choice options. As decision makers are often faced with decisions between exploration and exploitation, variations in risk signals could also influence these decisions by lending higher values to exploration or exploitation in risk seekers and avoiders. Thus, variations in risk signals between individuals could help to explain the familiar individual variations in subjective perceptions of risk and overt choice behaviour in the face of risky outcomes.

Our studies revealed neural signals differentiating between different degrees of uncertainty. This result appears to be incompatible with simplistic theories of decision making which postulate a similar impact of risk and ambiguity on choice behaviour. There were potentially two forms of neural distinction between risk and ambiguity. The striatum, parietal cortex and parts of frontal cortex encoded risk and ambiguity differentially according to a scheme of double dissociation. By contrast, other parts of frontal cortex and the amygdala showed stronger signals for ambiguous compared with risky gambles, suggesting graded coding of uncertainty as a quantitative continuum between risk (all probabilities known, lower signal) and full ambiguity (no probabilities known, higher signal). The graded coding of uncertainty may reflect unified neural treatment of risk and ambiguity as limiting cases of a general system evaluating uncertainty. For this hypothetical neural mechanism to have an impact on choice behaviour, ambiguity might be combined with expected value and integrated into expected utility in a similar way as risk, although the influence would be stronger. With this mechanism, risk-averse individuals would experience a stronger loss of expected utility with ambiguous compared with risky outcomes, which is frequently observed in overt choice behaviour.

The described outcome uncertainty signals occurred largely in brain structures that constitute foremost components of the brain's reward system, including the striatum, OFC, midbrain and amygdala. Both risk and value signals were seen in the striatum, although they differed in time course and regional location within the striatum (Preuschoff *et al*. 2006). Some of these differences may be due to the functional heterogeneity of inputs to the striatum, such as dopamine afferents, or local neurons in the striatum. Human imaging signals derive from large numbers of neurons and reveal only the strongest common signals while neglecting contributions from more dispersed functional groups. Thus, it remains to be seen whether separate striatal territories subserve risk and value or whether neurons coding these two parameters are intermingled.

The orbitofrontal activations with risk and ambiguity correspond to the deficits in decision making in the Iowa gambling task induced by orbitofrontal lesions (Bechara *et al*. 1994, 2000; Mobini *et al*. 2002; Sanfey *et al*. 2003), which occur with ambiguous outcomes during initial learning and risky outcomes after learning the probabilities. However, deficits in the Iowa gambling task may also relate to behavioural flexibility, reversal learning and attention shifting rather than misperceptions of risk *per se* (Maia & McClelland 2004; Dunn *et al*. 2006). Our findings may also help to explain the altered orbitofrontal activations during risky decisions in drug addicts (Bolla *et al*. 2005; Ersche *et al*. 2005).

## Figures and Tables

**Figure 1:**
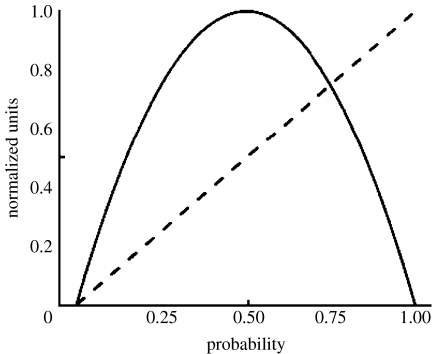
Expected reward and risk as a function of the probability of reward. Expected reward, measured as mathematical expectation of reward, increases linearly with the probability of reward *p* (dashed line). Expected reward is minimal at *p*=0 and maximal at *p*=1. Risk, measured as reward variance (or as its square root, standard deviation), follows an inverted U function of probability and is minimal at *p*=0 and 1 and maximal at *p*=0.5 (solid curve). Reprinted with permission from Preuschoff *et al*. (2006). Copyright © Cell Press.

**Figure 2:**
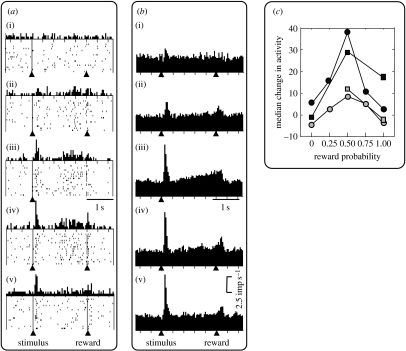
Risk signal in dopamine neurons. (*a*) Phasic reward value signal reflecting reward prediction (left) and more sustained risk signal during the stimulus–reward interval in a single dopamine neuron. Visual stimuli predicting reward probabilities (i) 0.0, (ii) 0.25, (iii) 0.5, (iv) 0.75 and (v) 1.0 alternated semi-randomly between trials. Both rewarded and unrewarded trials are shown at intermediate probabilities; the longer vertical marks in the rasters indicate delivery of the juice reward. (*b*) Population histograms of responses shown in (*a*). Histograms were constructed from every trial in 35–44 neurons per stimulus type (638 total trials at *p*=0 and 1200–1700 trials for all other probabilities). Both rewarded and unrewarded trials are included at intermediate probabilities. (i) 0.0, (ii) 0.25, (iii) 0.5, (iv) 0.75 and (v) 1.0. (*c*) Median sustained risk-related activation of dopamine neurons as a function of reward probability. Plots show the sustained activation as inverted U function of reward probability, indicating relationship to risk as opposed to value. Data from different stimulus sets and animals are shown separately. Reprinted with permission from Fiorillo *et al*. (2003). Copyright © American Association for the Advancement of Science.

**Figure 3:**
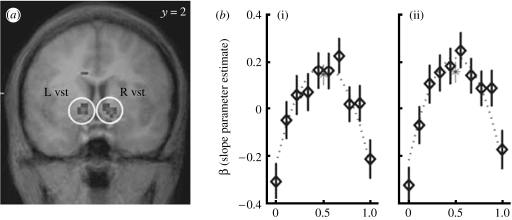
Risk signals in human ventral striatum. (*a*) Sustained BOLD response during 6 s correlated with variance as inverted U function of all-or-none reward probability (random effects, *p*<0.001; L vst, R vst for left, right ventral striatum). (*b*) Mean activations (parameter estimates beta with standard error) for 10 probabilities. Neural responses in striatum increased towards intermediate probabilities and decreased towards lower and higher probabilities. (i) Left vst and (ii) right vst. Dotted lines indicate best fit (*r*^2^=0.88–0.89, *p*<0.001). Grey data points at *p*=0.5 indicate late-onset activation between bet and first card when risk is maximal (*p*=0.5). Error bars=standard error of the mean (s.e.m). Reprinted with permission from Preuschoff *et al*. (2006). Copyright © Cell Press.

**Figure 4:**
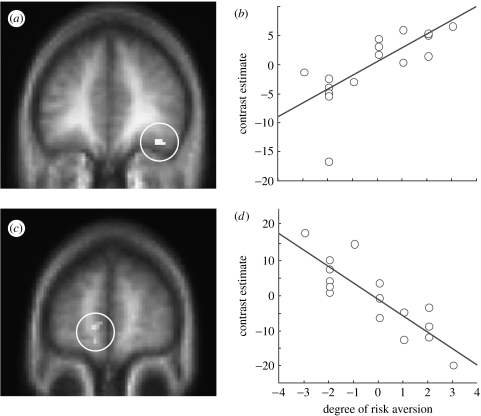
Relation of human orbitofrontal risk signals to individual risk attitude. (*a*, *b*) Risk signal in lateral OFC covarying with increasing risk aversion across participants (e.g. a ‘safety’ or ‘fear’ signal). (*b*) Correlation of contrast estimates of individual participants with their individual risk aversion (*p*<0.001, *r*=0.74; unpaired *t*-test in seven risk seekers and six risk averters). (*c*, *d*) Risk signal in medial OFC covarying with risk seeking (=inverse relation to risk aversion; e.g. a ‘risk seeking’ or ‘gambling’ signal). (*d*) Risk correlation analogous (*r*=0.85, *p*<0.0001) to (*b*). Abscissae in (*b*, *d*) show risk aversion as expressed by preference scores (−4 most risk seeking, +4 most risk aversion). To obtain these graphs, we correlated risk-related BOLD responses to individual risk attitude in two steps. First, we determined in each participant the contrast estimates reflecting the goodness of fit between brain activation and risk (variance as inverted U function of probability). Then, we regressed the contrast estimates of all participants to their individual behavioural risk preference scores and identified brain areas showing positive (*a*) or negative correlations (*c*). We plotted the regressions of risk aversion against the contrast estimates in (*b*, *d*). Reprinted with permission from Tobler *et al*. (2007). Copyright © The American Physiological Society.

**Figure 5:**
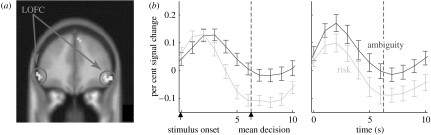
Ambiguity signals in human OFC. (*a*) Higher BOLD responses in OFC regions to stimuli-predicting ambiguous outcomes compared with risky outcomes, as identified by random effects analysis (*p*<0.001, uncorrected; 10 voxels; mean from card deck, knowledge and informed opponent situations). (*b*) Mean time courses of orbitofrontal BOLD responses to onset of stimuli-predicting ambiguous or risky outcomes (dashed vertical lines are mean decision times; error bars=standard error of the mean, s.e.m.; *n*=16 participants). (i) Left OFC and (ii) right OFC. Reprinted with permission from Hsu *et al*. (2005). Copyright © American Association for the Advancement of Science.
